# Facilitating islet transplantation using a three-step approach with mesenchymal stem cells, encapsulation, and pulsed focused ultrasound

**DOI:** 10.1186/s13287-020-01897-z

**Published:** 2020-09-18

**Authors:** Mehdi Razavi, Tanchen Ren, Fengyang Zheng, Arsenii Telichko, Jing Wang, Jeremy J. Dahl, Utkan Demirci, Avnesh S. Thakor

**Affiliations:** 1 Department of Radiology, Interventional Regenerative Medicine and Imaging Laboratory, Stanford University School of Medicine, 3155 Porter Drive, Palo Alto, CA 94304 USA; 2 Biionix™ (Bionic Materials, Implants & Interfaces) Cluster, Department of Internal Medicine, College of Medicine, University of Central Florida, Orlando, FL 32827 USA; 3 Department of Materials Science and Engineering, University of Central Florida, Orlando, FL 32816 USA; 4 Department of Radiology, Bio-Acoustic MEMS in Medicine Laboratory (BAMM), Stanford University School of Medicine, Palo Alto, CA 94304 USA; 5 Department of Radiology, Dahl Ultrasound Laboratory, Stanford University School of Medicine, Palo Alto, CA 94304 USA

**Keywords:** Islets transplantation, Mesenchymal stem cells, Encapsulation, Pulsed focused ultrasound, Diabetes

## Abstract

**Background:**

The aim of this study was to examine the effect of a three-step approach that utilizes the application of adipose tissue-derived mesenchymal stem cells (AD-MSCs), encapsulation, and pulsed focused ultrasound (pFUS) to help the engraftment and function of transplanted islets.

**Methods:**

In step 1, islets were co-cultured with AD-MSCs to form a coating of AD-MSCs on islets: here, AD-MSCs had a cytoprotective effect on islets; in step 2, islets coated with AD-MSCs were conformally encapsulated in a thin layer of alginate using a co-axial air-flow method: here, the capsule enabled AD-MSCs to be in close proximity to islets; in step 3, encapsulated islets coated with AD-MSCs were treated with pFUS: here, pFUS enhanced the secretion of insulin from islets as well as stimulated the cytoprotective effect of AD-MSCs.

**Results:**

Our approach was shown to prevent islet death and preserve islet functionality in vitro. When 175 syngeneic encapsulated islets coated with AD-MSCs were transplanted beneath the kidney capsule of diabetic mice, and then followed every 3 days with pFUS treatment until day 12 post-transplantation, we saw a significant improvement in islet function with diabetic animals re-establishing glycemic control over the course of our study (i.e., 30 days). In addition, our approach was able to enhance islet engraftment by facilitating their revascularization and reducing inflammation.

**Conclusions:**

This study demonstrates that our clinically translatable three-step approach is able to improve the function and viability of transplanted islets.

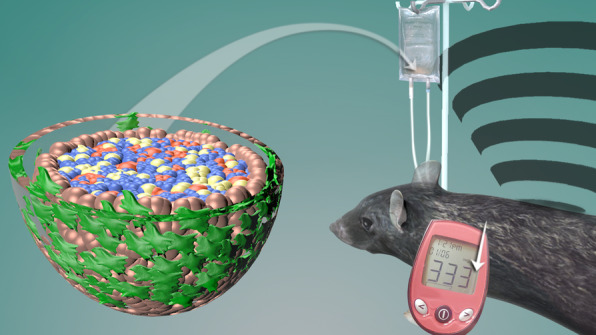

## Introduction

Type 1 diabetes (T1D) affects more than 1.5 million individuals in the USA and 20 million worldwide [1]. T1D is a chronic autoimmune disease caused by the selective destruction of insulin producing β cells within pancreatic islets resulting in patients requiring exogenous insulin to maintain blood glucose control [2]. One solution to restore glycemic control in patients with T1D is pancreatic islet transplantation whereby healthy donor islets are infused into the liver of a patient with T1D [3, 4]. However, over 60% of islets are lost in the immediate period following transplantation as a result of the instant blood-mediated inflammatory reaction (IBMIR) towards islets, as well as the lack of a dedicated blood supply to islets given that they get devascularized during their isolation procedure [5].

One strategy to improve islet survival and engraftment following transplantation is to co-transplant them with mesenchymal stem cells (MSCs). MSCs are self-renewable, multi-potent non-hematopoietic progenitor cells that are ubiquitously found in a number of tissues throughout the body, including adipose tissue (AD-MSCs). MSCs can secrete soluble trophic factors (i.e., angiogenic, anti-inflammatory, anti-apoptotic, immunomodulatory and anti-fibrotic factors [6, 7]) into their surrounding microenvironment that can modulate the immune system and stimulate the endogenous regeneration of damaged tissues [8]. Interestingly, the culture medium collected from MSCs has been shown to contain soluble factors that can orchestrate interactions within the microenvironment to facilitate tissue regeneration, thereby suggesting that the protective and regenerative effects of MSCs are predominantly mediated via paracrine actions. Hence, MSCs appear to be an ideal candidate to be co-transplanted with islets, given that they could help islets establish their own vasculature (via angiogenic factors) and protect islets from the IBMIR and any toxicity-related issues related to immunosuppressive medications (via anti-inflammatory and immunomodulatory factors). Furthermore, recent studies have shown that AD-MSCs can increase islet survival and function, in vitro as well as in vivo following transplantation [9–12].

In the clinical setting, when MSCs have been co-transplanted with pancreatic islets, the MSCs were administered into the liver *after* the islets had been infused [13]. Given the large volume of the liver, and the anatomical branching pattern of the portal vein (i.e., the vessel in which both islets and MSCs are infused into), it is almost impossible to ensure that MSCs would be spatially located next to islets using this approach. Hence, for MSCs to be effective, they need to be in close proximity to islets to both sample the surrounding microenvironment as well as then release the appropriate paracrine factors, which can then reach and help the transplanted islets. One way to ensure that MSCs are “spatially coupled” next to islets at the time of transplantation is to encapsulate them together. This approach will ensure that each islet will have its own cohort of MSCs within its immediate proximity, thereby enhancing their protective and supportive effects on islets. In the present study, we therefore used a high-throughput, reproducible, and scalable co-axial airflow technique to conformally encapsulate islets and MSCs with an ultrapure formulation of alginate. Given that the alginate capsule is semi-permeable and thin (i.e., conformal coating ranges from 50 to 100 μm [14, 15]), it will allow nutrients, oxygen, and glucose to diffuse to islets while concurrently enabling waste products to diffuse away from islets [16]. Furthermore, it also provides a physical barrier around islets to protect them from any immune mediated attack [17].

**Fig. 1:**
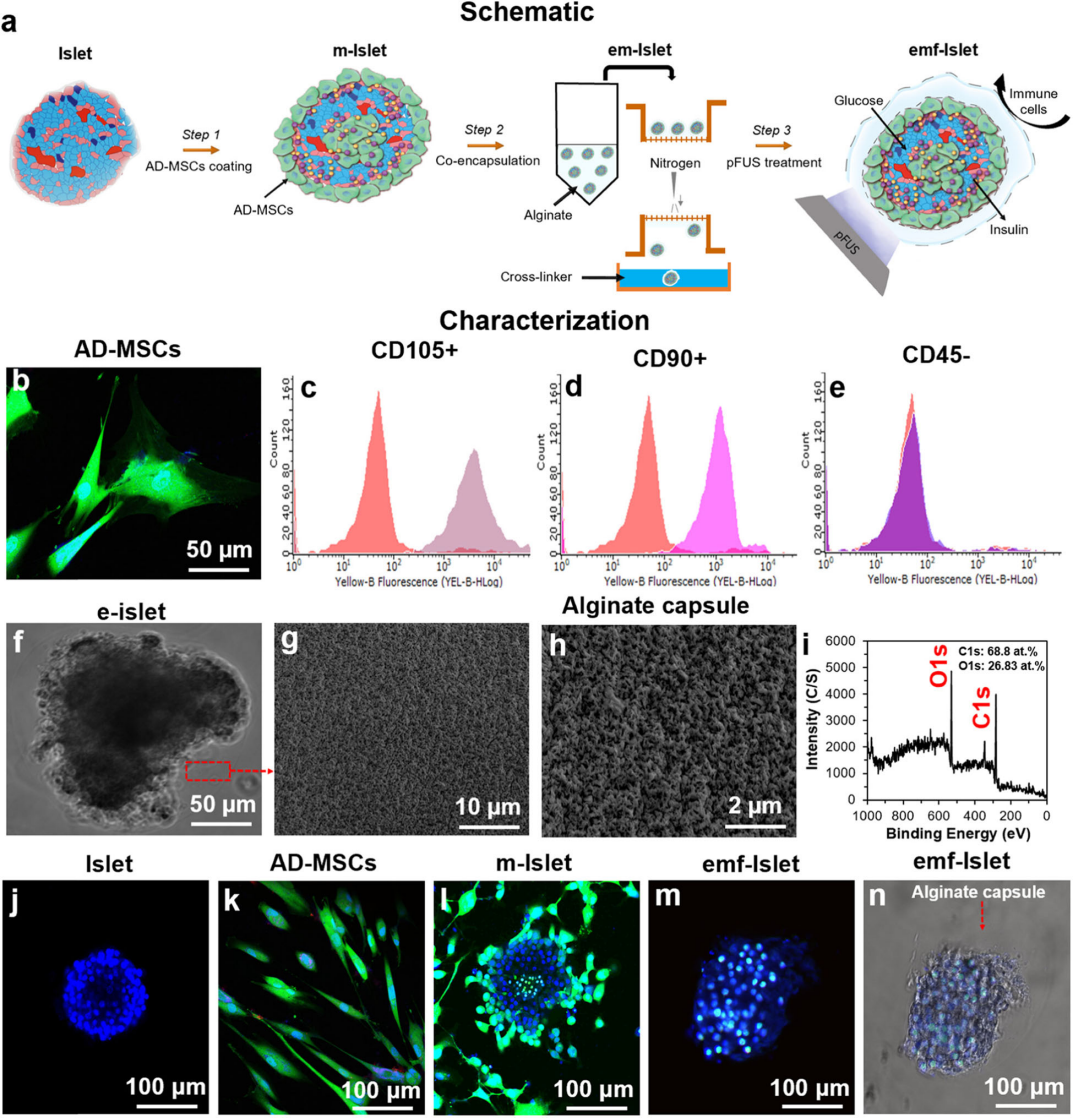
Experimental overview and characterization of islets encapsulated with AD-MSCs: **a** Schematic representation of our three-step approach: step 1: AD-MSC coating, step 2: encapsulation, and step 3: pFUS treatment; characterization of **b**–**e** AD-MSCs (**b** confocal image and **c**–**e** FACS analysis), **f**–**i** alginate capsule (photographic, SEM images, and XPS scans), and **j**–**n** confocal images of an **j** islet, **k** AD-MSCs, **l** islet coated with AD-MSCs, and **m**–**n** encapsulated with alginate followed by pFUS treatment. Blue: live cells stained with Hoechst. Green: AD-MSCs stained with FDA

However, once islets and MSCs have been administered into patients, there is currently no existing method to non-invasively stimulate either of them in vivo. One interesting solution to this problem is to sonicate them with sound waves. Focused ultrasound (FUS) is a novel technology, available at many institutions across the world, which can focus sound waves at specific locations deep in the body, with pin-point accuracy, without the use of any incisions. Pulsed focused ultrasound (pFUS) is a variation of this technology that uses short duty-cycles to minimize temperature elevations, thereby allowing the mechanical effects of ultrasound to predominate [18]. We have recently shown that islets treated with pFUS were stimulated resulting in an increase in their function. This improvement in islet function was a result of pFUS increasing the intracellular concentration of calcium (Ca^2+^) within islets which was also linked to pFUS increasing the resting membrane potential (*V*_*m*_) of islets [19]. Potentially, pFUS would enable, for the first time, a completely non-invasive approach to rescue struggling islets and/or stimulate the regenerative function of MSCs, after these cells have been delivered into patients. Hence, the present study examined the effect of pFUS on helping the engraftment and function of islets encapsulated with AD-MSCs (Fig. 1a). We used an STZ-induced diabetic mouse model and the kidney capsule was chosen as the site of transplantation given that it is a well-established and accessible site for islet transplantation in small animals [20–22].

## Materials and methods

### Islet and AD-MSC isolation and characterization

All mice in this study were treated in accordance with the guidelines approved by the Institutional Animal Care and Use Committee (IACUC) at Stanford University. Animals were housed under conventional conditions having access to food and water ad libitum. Pancreatic islets were isolated from C57/B6 mice (male, 6–8 week-old, Charles River Laboratories, USA), as previously described (see Supplemental Information) [23]. AD-MSCs were also obtained from the mouse adipose tissue of male C57BL/6 mice at 6–8 weeks of age and characterized as previously described (see Supplemental Information) [24].

### Step 1: AD-MSC coating on islets

In a 50-mm low-adherence culture dish (Corning, USA), 500 islets and 250,000 AD-MSCs (i.e., islet: AD-MSC ratio of 1:500) were added and gently mixed together by pipetting up-and-down 5x before being incubated for 24 h at 37 °C and 5% CO_2_. These parameters were chosen to ensure islets were optimally coated with AD-MSCs [25]. Islets coated with AD-MSCs were then manually picked under a bright-field microscope (the percentage of islets coated with AD-MSCs was > 95%) and transferred into a 15-mL falcon tube where they were allowed to settle for 0.5 h at 37 °C and 5% CO_2_ before the supernatant was decanted.

### Step 2: Encapsulation of islets coated with AD-MSCs

Encapsulation of islets coated with AD-MSCs was performed by suspending the cell pellet in a sodium alginate solution (2 wt.%, Sigma Aldrich, USA) containing mannose (1 wt.%, Sigma Aldrich, USA) in 4-(2-hydroxyethyl)-1-piperazineethanesulfonic acid (HEPES; 10 mM, ThermoFisher Scientific, USA). The solution was transferred to a cell strainer (70 μm, Fischer Scientific, USA) to collect the encapsulated islets. The cell strainer was then inverted in order to spray these encapsulated cells into a CaCl_2_ (150 mM, Sigma Aldrich, USA) solution which contained a surfactant—pluronic F-127 (0.04 wt.%, Sigma Aldrich, USA) in HEPES (10 mM, ThermoFisher Scientific, USA). Pressurized ultrapure nitrogen (speed: 2 mm^3^/s) was then used to spray the encapsulated cells out of the cell strainer. The synthesized alginate capsules were then characterized (see Supplemental Information).

### Step 3: pFUS treatment on encapsulated islets coated with AD-MSCs

#### In vitro

Details of the pFUS set-up, calibration, and output characterizations are described in the Supplemental Information. For each pFUS treatment, experiments were performed using a 12 well-plate (Corning, USA) containing 100 islets/well. Given that the ultrasound beam width (16 mm) was close to the diameter of an individual well, this allowed the simultaneous sonication of all the islets since they were predominantly seeded in the center of a well. Ultrasound gel was applied on the surface of the piston transducer to couple it with the bottom of the well plate. For the in vitro experiments, the following pFUS parameters were used: 1 MHz frequency, 2000 cycles per pulse, with a pulse repetition frequency (PRF) of 100 Hz, 20% duty cycle (DC), 150 kPa peak negative pressure (PNP), 1.43 W/cm^2^ spatial average pulse average intensity (I_sptp_), and 1 min exposure time. The selection of our pFUS parameters was based on previous literature showing that these parameters could improve cellular function with no adverse effect on cell growth and/or viability [26, 27].

#### In vivo

Islets were transplanted under the kidney capsule of diabetic mice (see In vivo analysis of islet survival and function). Transplanted islets were then treated with pFUS in vivo as described in the Supplemental Information. To treat the whole kidney, 8 non-overlapping adjacent regions through the kidney were targeted for 30 sec per region. The time to treat one kidney with these parameters was approximately 4 min. In order to deliver pFUS therapy to the animal, the following pFUS parameters were used: 5 Hz PRF, 5% DC, 2.9 MPa PNP, and 895 W/cm^2^ I_sptp_, which has been shown in previous studies to be safe in small animals [28]. After pFUS treatment, each mouse was removed from the water bath, dried, and placed in a recovery cage.

Our experimental groups include islets only, encapsulated islets only, islets coated with AD-MSCs, encapsulated islets coated with AD-MSCs, and encapsulated islets coated with AD-MSCs followed by pFUS treatment; for clarity, these will be called throughout the manuscript as the following: Islets, e-Islets, m-Islets, em-Islets, and emf-Islets, respectively.

### In vitro analysis of islet survival and function

There were 4 experimental groups tested: group 1 = Islets (*n* = 5; control group); group 2 = m-Islets (*n* = 5; step 1); group 3 = em-Islets (*n* = 5; step 2); group 4 = emf-Islets (*n* = 5; step 3). MTT, live/dead, and glucose-stimulated insulin secretion (GSIS) assays were performed as described in the Supplemental Information. Each experiment contained 30 islets in a 96 well plate (30 islets/well). As required, pFUS-treated islets were selected from a 12-well plate which contained 100 islets per well.

### In vivo analysis of islet survival and function

There were 5 experimental groups tested: group 1 = mice transplanted with Islets (*n* = 5; control group), group 2 = mice transplanted with e-Islets (*n* = 5; control group), group 3 = mice transplanted with m-Islets (*n* = 5; step 1), group 4 = mice transplanted with em-Islets (*n* = 5; step 2), and group 5 = mice transplanted with emf-Islets (*n* = 5; step 3). All experiments were approved by the Institutional Animal Care and Use Committee (IACUC) at Stanford University. Male C57BL/6 mice, at 6–8 weeks age (Charles River Laboratories, USA), were used as both donor and recipient animals. All animals were maintained on a 12 h:12 h light to dark cycle with ad libitum access to food and water. Recipient mice were matched for their body weight and baseline blood glucose levels. Prior to islet transplantation, all recipient mice were made diabetic (i.e., determined by 2 consecutive non-fasting blood glucose levels > 350 mg/dl, as previously documented [29]) by an intraperitoneal injection of streptozotocin (STZ; 180 mg/kg). Each diabetic mouse then received 175 handpicked islets under the right kidney capsule before being randomly allocated to an experimental group. Mice transplanted with em-Islets were then treated with pFUS at days 3, 6, 9, and 12 post-transplantation (see **Step 3: pFUS treatment on encapsulated islets coated with AD-MSCs**). Experimental details of our in vivo experiment are outlined in Fig. 4a. Metabolic, histological, and molecular analyses were then performed as described in Supplemental Information.

### Statistical analysis

All values were expressed as the mean ± standard error of the mean (SEM). Statistical analysis of all quantitative data was performed using a one or two-way ANOVA (analysis of variance) with post hoc Tukey test (Astatsa.com; Online Web Statistical Calculators, USA) or unpaired Student’s *t* test with any differences considered statistically significant when *P* < 0.05.

## Results

### Characterizations

AD-MSCs had a long and thin morphology with widely dispersed filopodia and flattened polygonal extensions (Fig. 1b). Analysis of surface antigen expression showed that AD-MSCs expressed CD105 (90.53 ± 5.45) and CD90 (92.41 ± 3.62) markers (positive) with no expression of the CD45 (2.43 ± 0.72) marker (negative) (Fig. 1c–e). Characterization studies showed that alginate capsule had a porous structure with the pore size of 200 ± 50 nm (Fig. 1f–h). In XPS spectra, alginate showed peaks corresponding to the elements of carbon (C) and oxygen (O) which are the basic elements of alginate (Fig. 1i). When islets were co-cultured with AD-MSCs with the ratio of 1:500 for 24 h, AD-MSCs attached to islets and coated their surfaces. Following encapsulation, an alginate layer in a 50 ± 10 μm thick was formed on m-Islets. Results of confocal imaging confirmed that pFUS treatment did not adversely affect islets quality since islets could maintain their spherical shape and kept their integrity (Fig. 1j–n).

### In vitro analysis of islet survival and function

Each mouse islet is made up of a central core of beta cells, surrounded by a mantle of alpha, delta, epsilon, and pancreatic polypeptide cells [30]. The beta cell produces the hormone insulin and makes up approximately 75% of each islet [31]. Compared to alpha, delta, epsilon, and pancreatic polypeptide cells, beta cells are very susceptible to inflammatory proteins (cytokines). Furthermore, inflammation can cause beta cells to present themselves as targets of the immune system, enhancing the T cell attack that kills them [32]. Hence, dead cells (shown by the red color) seen in the center of islets in our confocal images are representative of beta cells. The live cells (shown by the blue color) in the periphery of the islets are likely the other cells types mentioned above, predominantly consist of alpha cells [33].

#### Step 1: AD-MSC coating on islets

**Fig. 2:**
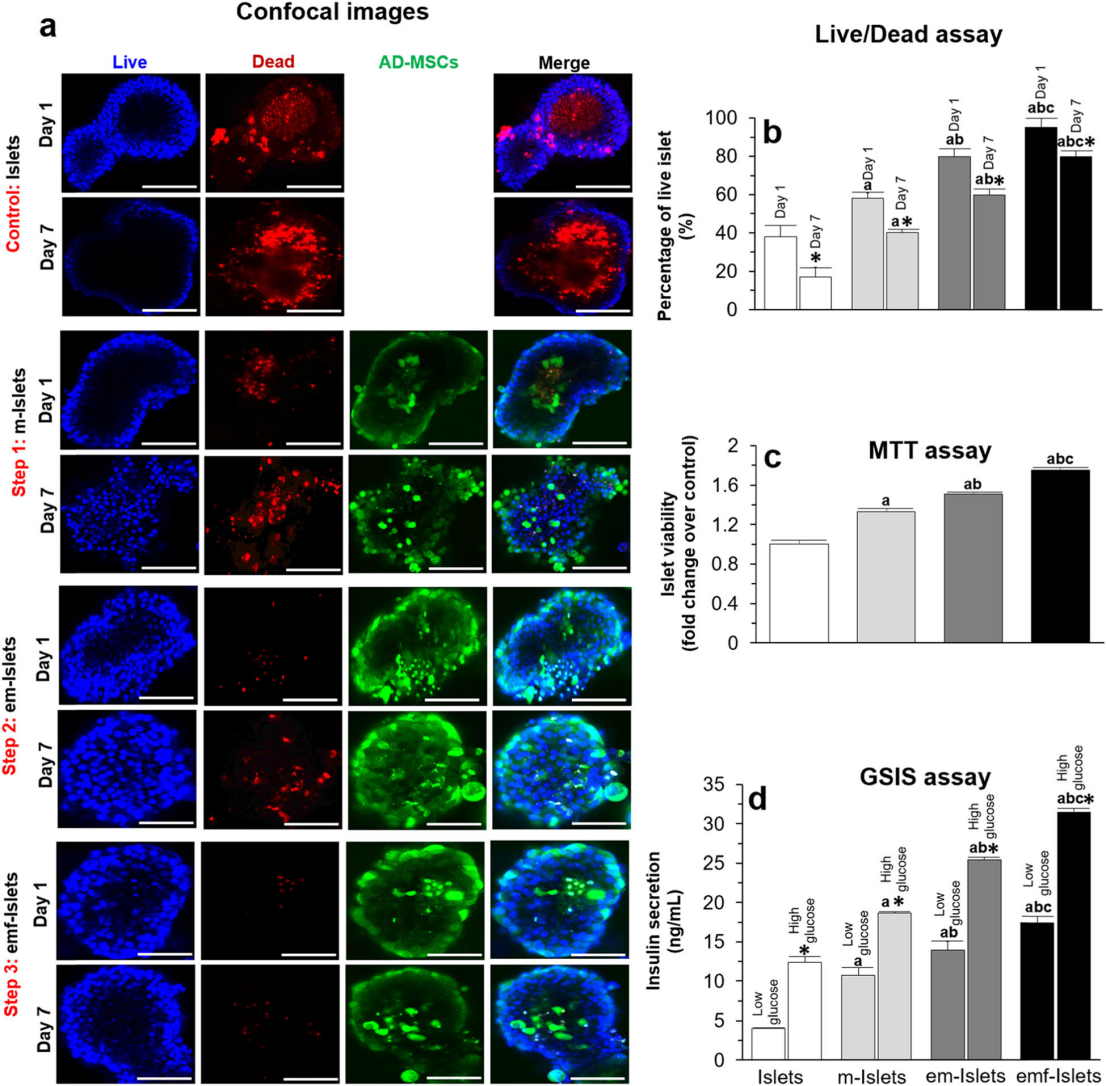
In vitro analysis of islet survival and function in normal conditions: **a** Representative confocal images, and results of **b** live/dead, **c** MTT, and **d** GSIS assays of tested groups (i.e., Islets, e-Islets, m-Islets, em-Islets, and emf-Islets). Confocal images and live/dead assay have been performed at days 1 and 7, and MTT and GSIS assays at day 7, in culture. Blue: live cells stained with Hoechst. Red: dead cells stained with PI. Green: AD-MSCs stained with FDA. Scale bar = 50 μm. Significant differences: **b**–**d** ^a^*P* < 0.05: Islets vs. m-Islets or em-Islets or emf-Islets; ^b^*P* < 0.05: m-Islets vs. em-Islets or emf-Islets; ^d^*P* < 0.05: em-Islets vs. emf-Islets (one-way ANOVA post hoc Tukey test). **b** *P < 0.05: day 1 vs. day 7; **d** *P < 0.05: high glucose vs. low glucose (two (**b**, **d**) or one (**c**)-way ANOVA post hoc Tukey test)

Results of live/dead assay at days 1 and 7 showed that the percentage of live islets was 38 ± 6 and 17 ± 5%, respectively for islet only. When islets were coated with AD-MSCs, islet survival significantly increased compared to Islets at days 1 (58 ± 3 vs. 38 ± 6%, *P* < 0.05) and 7 (40 ± 2 vs. 17 ± 5%, *P* < 0.05; Fig. 2a, b). MTT assay results, at day 7, relative to Islets, demonstrated that there was a significantly greater viability of islets when they were coated with AD-MSCs and encapsulated (1.50 ± 0.02 vs. 1.00 ± 0.03 fold change, *P* < 0.05; Fig. 2v). Furthermore, em-Islets had a significantly higher viability compared to m-Islets (1.50 ± 0.02 vs. 1.33 ± 0.03, *P* < 0.05; Fig. 2c). Using GSIS assay, islets indicated more responsiveness to high glucose challenge when they were coated with AD-MSCs compared to Islets (18.68 ± 0.15 vs. 12.36 ± 0.77 ng/mL, *P* < 0.05, Fig. 2d).

#### Step 2: Encapsulation of islets coated with AD-MSCs

Encapsulation of islet coated with AD-MSCs significantly altered islet viability post-encapsulation where the percentage of live islet was significantly higher compared to m-Islets and Islets (80 ± 4 vs. 58 ± 3 and 38 ± 6% at day 1 and 85 ± 1 vs. 40 ± 2 and 17 ± 5% at day 7, *P* < 0.05; Fig. 2a, b). MTT assay results, at day 7, relative to Islets, demonstrated that there was a significantly greater viability of islets when they were coated with AD-MSCs and encapsulated (1.50 ± 0.02 vs. 1.00 ± 0.03 fold change, *P* < 0.05; Fig. 2c). At day 7, em-Islets had a greater insulin secretory response to a high glucose challenge compared to m-Islets and Islets (25.46 ± 0.32 vs. 18.68 ± 0.15 and 12.36 ± 0.77 ng/mL, respectively, *P* < 0.05; Fig. 2d).

#### Step 3: pFUS treatment on encapsulated islets coated with AD-MSCs

When em-Islets were treated with pFUS, the amount of live cells significantly increased to 95 ± 5 and 80 ± 3% at days 1 and 7, respectively (*P* < 0.05; Fig. 2a, b). MTT assay results showed that, at day 7, relative to Islets, there was a significantly greater viability of islets when they were coated with AD-MSCs, encapsulated, and also treated with pFUS (Fig. 2c; 1.75 ± 0.03 vs. 1.00 ± 0.03 fold change, *P* < 0.05). Using a GSIS assay, islets were more responsive to glucose challenges when they were treated with pFUS. Compared to Islets, there was a significantly greater function of islets when they were coated with AD-MSCs, encapsulated and also treated with pFUS. At day 7, em-Islets which had been treated with pFUS had a greater insulin secretory response to high glucose challenges compared to Islets, m-Islets, and em-Islets (31.56 ± 0.45 vs. 12.36 ± 0.77, 18.68 ± 0.15, and 25.46 ± 0.32 ng/mL, respectively, *P* < 0.05; Fig. 2d).

**Fig. 3:**
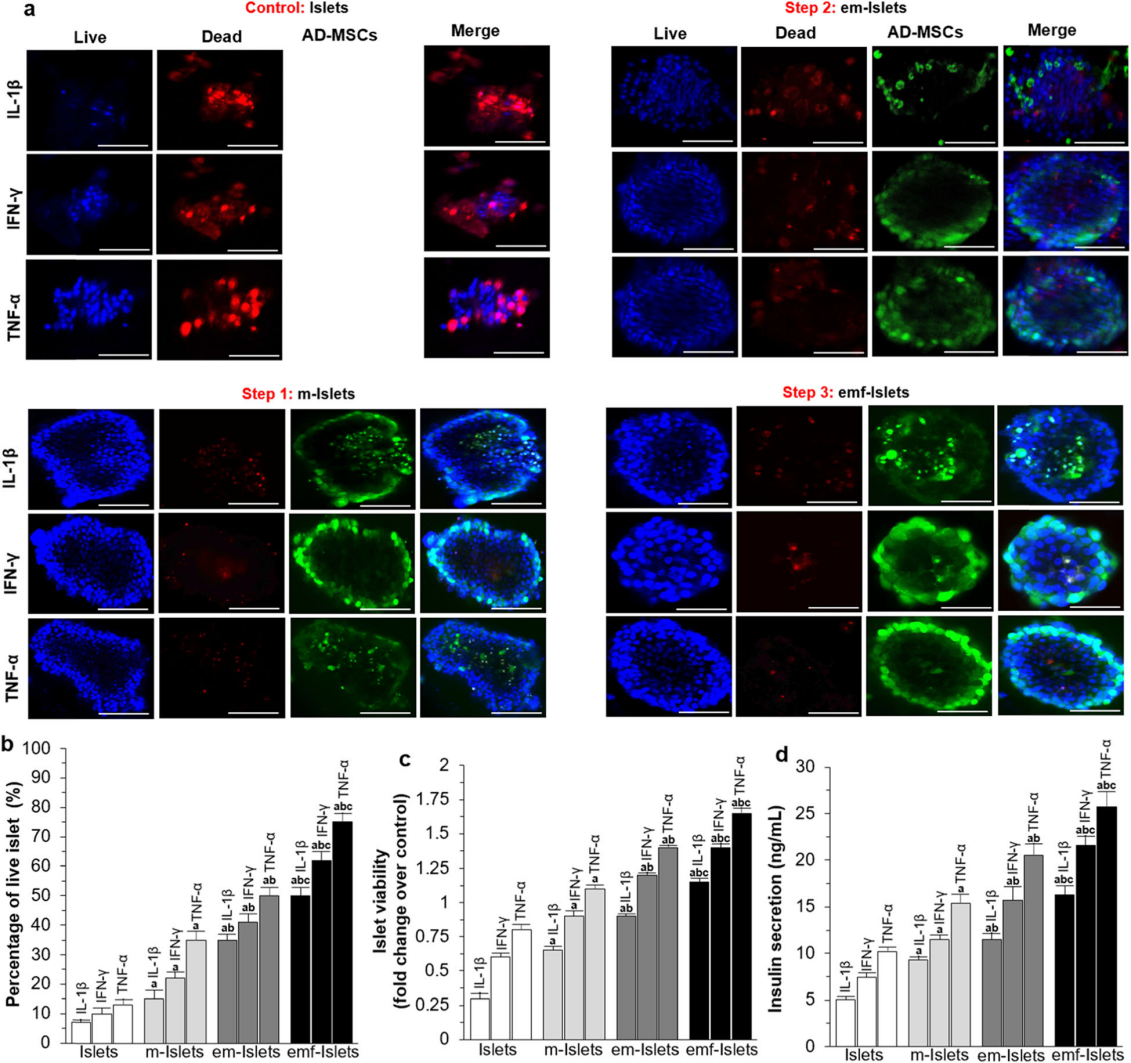
In vitro analysis of islet survival and function following exposure to pro-inflammatory cytokines: **a** representative confocal images and results of **b** live/dead, **c** MTT, and **d** high-glucose-stimulated insulin secretion assays of tested groups (i.e., Islets, e-Islets, m-Islets, em-Islets, and emf-Islets). Blue: live cells stained with Hoechst. Red: dead cells stained with PI. Green: AD-MSCs stained with FDA. Scale bar = 50 μm. Significant differences: **b**–**d** ^a^*P* < 0.05: Islets vs. m-Islets or em-Islets or emf-Islets; ^b^*P* < 0.05: m-Islets vs. em-Islets or emf-Islets; ^d^*P* < 0.05: em-Islets vs. emf-Islets (one-way ANOVA post hoc Tukey test)

After exposure to pro-inflammatory cytokines including IL-1β or IFN-γ or TNF-α, the percentage of live islets at day 7 was 7 ± 1, 10 ± 2, and 13 ± 2%, respectively, for Islets. When islets were coated with AD-MSCs, encapsulated in alginate, and treated with pFUS, they were able to maintain a higher level of viability compared to islets only at day 7 following exposure to IL-1β (15 ± 3, 35 ± 2, 50 ± 3 vs. 7 ± 1%, *P* < 0.05), IFN-γ (22 ± 2, 41 ± 3, 62 ± 3 vs. 10 ± 2%, *P* < 0.05), and TNF-α (35 ± 3, 50 ± 3, 75 ± 3 vs. 13 ± 2%, *P* < 0.05; Fig. 3a–e). Similar results were obtained with MTT assays (i.e., viability of m-Islets, em-Islets, and emf-Islets were significantly higher compared to Islets when islets exposed to IL-1β (0.65 ± 0.03, 0.90 ± 0.02, and 1.15 ± 0.03 vs. 0.30 ± 0.04 fold change over control, *P* < 0.05), IFN-γ (0.90 ± 0.04, 1.20 ± 0.02, and 1.40 ± 0.03 vs. 0.60 ± 0.03 fold change over control, *P* < 0.05), and TNF-α (1.10 ± 0.03, 1.40 ± 0.02, and 1.65 ± 0.04 vs. 0.80 ± 0.04 fold change over control, *P* < 0.05; Fig. 3f)). Following high-glucose-stimulated insulin secretion assays, the amounts of insulin secreted from Islets was 5.1 ± 0.3, 7.4 ± 0.5, and 10.2 ± 0.5 ng/mL when islets exposed to IL-1β, IFN-γ, and TNF-α, respectively. However, AD-MSC coating, co-encapsulation, and pFUS treatment significantly elevated the insulin level to 9.3 ± 0.4, 11.5 ± 0.7, and 16.3 ± 1 ng/mL, respectively, when islets exposed to IL-1β (*P* < 0.05), 11.5 ± 0.5, 15.7 ± 1.5, and 21.6 ± 1 ng/mL, respectively, when exposed to IFN-γ (*P* < 0.05), and 15.4 ± 1, 20.5 ± 1.3, and 25.7 ± 1.7 ng/mL, respectively, when exposed to TNF-α (*P* < 0.05).

### In vivo analysis of islet survival and function

#### Metabolic analysis

**Fig. 4:**
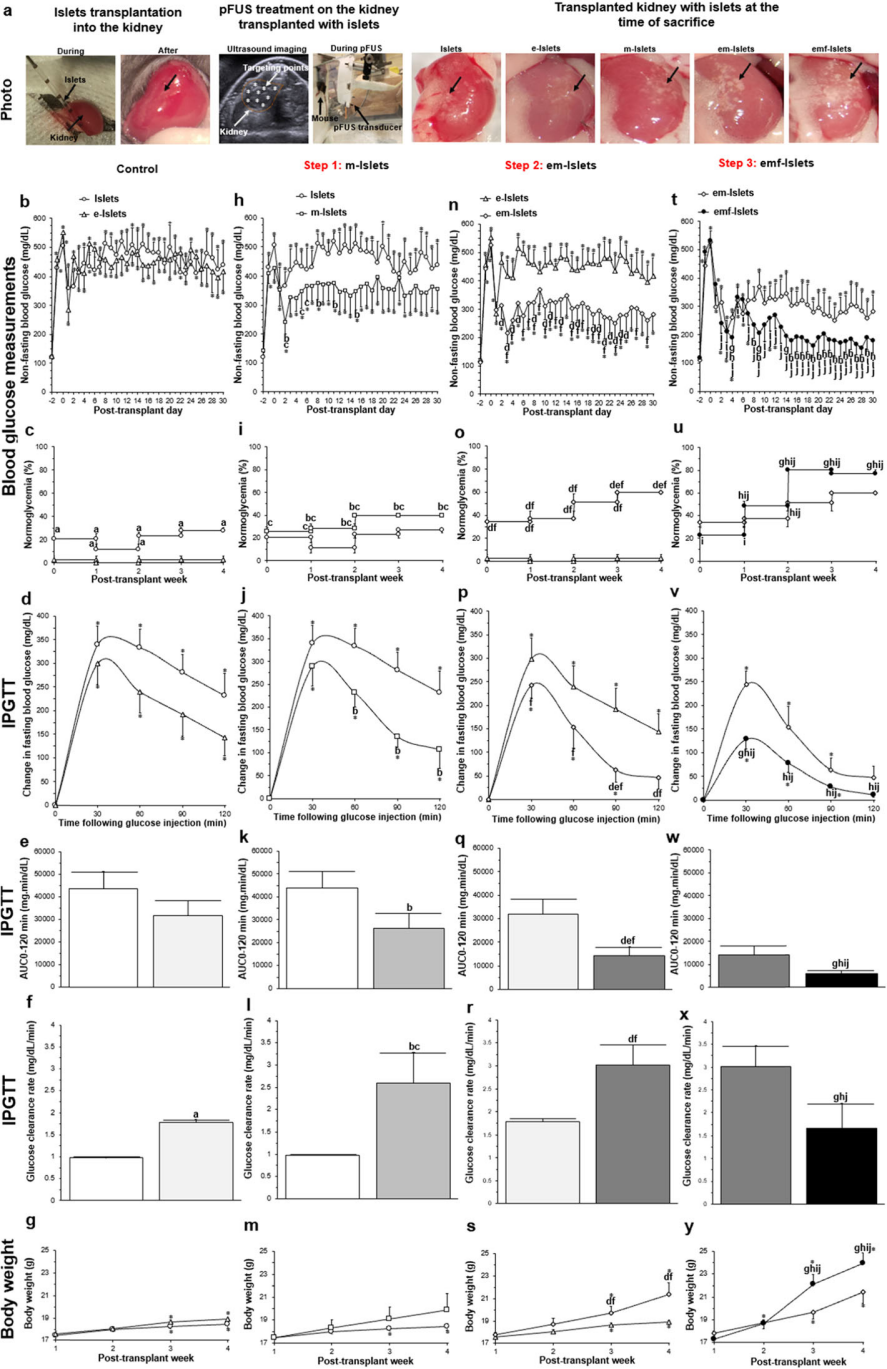
In vivo analysis of islet survival and function (metabolic analysis): **a** islet transplantation using a kidney subcapsular approach; pFUS treatment on the kidney transplanted with islets; transplanted kidney with different experimental groups (i.e., Islets, e-Islets, m-Islets, em-Islets, and emf-Islets) at the time of euthanasia (black arrows indicate transplanted islets); results of **b**–**e** BG measurements, **f**–**i** normoglycemia percentage, **j**–**m** IPGTT, **n**–**q** area under the IPGTT curve (AUC_0-120min_), **r**–**u** BG clearance rates calculated from slope of IPGTT curves from 30 to 90 min and **v**–**y** body weight of mice post-transplant measured at various time points over 30 days. Results shows the effect of **b**, **f**, **j**, **n**, **r**, **v** islet encapsulation by comparing Islets with e-Islets, **c**, **g**, **k**, **o**, **s**, **w** AD-MSC coating on islets by comparing Islets with m-Islets, **d**, **h**, **l**, **p**, **t**, **x** encapsulating islets with AD-MSCs by comparing e-Islets with em-Islets, **e**, **i**, **m**, **q**, **u**, **y** and pFUS treatment by comparing em-Islets with emf-Islets groups. Significant differences: **b**–**y** ^a^*P* < 0.05: e-Islets vs. Islets; ^b^*P* < 0.05: m-Islets vs. Islets; ^c^*P* < 0.05: m-Islets vs. e-Islets; ^d^*P* < 0.05: em-Islets vs. e-Islets; ^e^*P* < 0.05: em-Islets vs. m-Islets; ^f^*P* < 0.05: em-Islets vs. Islets; ^g^*P* < 0.05: emf-Islets vs. em-Islets; ^h^*P* < 0.05: emf-Islets vs. m-Islets; ^i^*P* < 0.05: emf-Islets vs. e-Islets; ^j^*P* < 0.05: emf-Islets vs. Islets; *P < 0.05: baseline vs. all other time-points (two (**b**, **h**, **n**, **t**, **d**, **j**, **p**, **v**, **g**, **m**, **s**, **y)** or one (**k**, **q**, **w**, **l**, **r**, **x**)-way ANOVA post hoc Tukey test or unpaired Student’s *t* test (**e**, **f**))

Following treatment with STZ, all animals became hyperglycemic with their blood glucose (BG) values increasing from 116 ± 10 mg/dL (baseline, day − 2) to 535 ± 20 mg/dL (post-STZ treatment, day 0; Fig. 4b, h, n, t). Reversal of hyperglycemia was observed immediately in all groups that received islet transplants. Comparing BG values at day 1 post-transplant, values for mice transplanted with e-Islets was similar to those mice transplanted with Islets (284 ± 42 vs. 382 ± 65 mg/dL, *P* > 0.05). This effect was sustained throughout the course of the study with mice transplanted with e-Islets having no significant difference in BG values from days 1 to 30 compared to mice transplanted with Islets (*P* > 0.05; Fig. 4b). The percentage of recipient mice which exhibited normoglycemia (normoglycemia%) in the first week following transplantation was 21 ± 5% for those which were transplanted with Islets; this decreased to 12 ± 5% at week 2 and then increased to 23 ± 3% at week 3 and 28 ± 3% at week 4 post-transplantation. In contrast, mice transplanted with e-Islets remained diabetic from week 1 to 4 (*P* < 0.05; Fig. 4c). Following intraperitoneal glucose tolerance tests (IPGTT), mice transplanted with e-Islets showed a similar peak value (*P* > 0.05; Fig. 4d) with no change in the area under the curve (AUC_0-120min_^,^ *P* > 0.05; Fig. 4e) when compared to mice transplanted with Islets. However, the BG clearance rate (calculated from slope of BG change vs. time from 30 to 90 min) was significantly higher for mice transplanted with e-Islets when compared to mice transplanted with Islets (*P* < 0.05; Fig. 4f). The body weight of all mice increased following transplantation. However, during the course of our study, the increase in body weight of mice transplanted with e-Islets was not significantly different when compared to mice transplanted with Islets (*P* > 0.05; Fig. 4g).

##### Step 1: AD-MSC coating on islets

At days 2, 8, 11, and 15 post-transplantation, BG values for mice transplanted with m-Islets was significantly lower than mice transplanted with Islets (*P* < 0.05). Compared to mice transplanted with e-Islets, at days 2, 5, and 6 post-transplantation, BG values for mice transplanted with m-Islets was significantly lower (*P* < 0.05). However, BG values from days 1 to 30 post-transplantation in mice transplanted with m-Islets were significantly higher than their own baseline values pre-transplantation (*P* < 0.05; Fig. 4h). The normoglycemia% for mice transplanted with m-Islets was significantly higher compared to mice transplanted with Islets (except at week 1) or e-Islets (*P* < 0.05; Fig. 4i). Following IPGTT, mice transplanted with m-Islets showed a significant decrease in BG values from 60 to 120 min when compared to mice transplanted with Islets (*P* < 0.05; Fig. 4j). This caused a significant reduction in the AUC_0-120min_ (*P* < 0.05; Fig. 4k) and an increase in BG clearance rate compared to mice transplanted with Islets (*P* < 0.05). Furthermore, compared to mice transplanted with e-Islets, the BG clearance rate of mice transplanted with m-Islets was significantly higher (*P* < 0.05; Fig. 4l). From week 1 to 4 post-transplantation, the body weight of mice transplanted with m-Islets increased; however, this increase was not significant (*P* > 0.05). Although the body weight of mice transplanted with m-Islets was higher compared to mice transplanted with Islets or e-Islets, these differences were also not significant (*P* > 0.05; Fig. 4m).

##### Step 2: Encapsulation of islets coated with AD-MSCs

At day 1 post-transplantation, BG values of mice transplanted with em-Islets was similar to mice transplanted with e-Islets (298 ± 72 vs. 284 ± 42 mg/dL, *P* > 0.05). However, at day 2 post-transplantation, BG values for mice transplanted with em-Islets significantly decreased compared to mice transplanted with e-Islets (313 ± 70 vs. 468 ± 19 mg/dL; *P* < 0.05). This effect was sustained throughout the course of our study with mice transplanted with em-Islets having significantly lower BG values from days 2 to 30 except at days 6, 7, 9, 12, 14, 17, 18, and 26–30 compared to mice transplanted with e-Islets (*P* < 0.05). Mice transplanted with em-Islets showed significantly lower BG values from days 2 to 30 except at days 1, 2, 5, 14, 22, 26, 28, and 30 compared to islets only. However, BG values in mice transplanted with em-Islets were still significantly higher compared to their own baseline values pre-transplantation (*P* < 0.05; Fig. 4n). The normoglycemia% for em-Islets was significantly higher compared to mice transplanted with Islets or e-Islets from weeks 1 to 4 and higher compared to mice transplanted with m-Islets at week 4 (*P* < 0.05; Fig. 4o). Following IPGTT testing, mice transplanted with em-Islets showed a significant decrease in BG values from 30 to 120 min compared to mice transplanted with Islets (*P* < 0.05; Fig. 4p). The AUC_0-120min_ for mice transplanted with em-Islets was significantly lower than mice transplanted with e-Islets or m-Islets or Islets (*P* < 0.05; Fig. 4q). Furthermore, mice transplanted with em-Islets had a significantly improved BG clearance rate compared to mice transplanted with Islets or e-Islets (*P* < 0.05; Fig. 4r). Comparing the body weight of mice transplanted with e-Islets or Islets, mice transplanted with em-Islets had a significantly higher body weight at week 3 (*P* < 0.05) and 4 (*P* < 0.05; Fig. 4s).

##### Step 3: pFUS treatment on encapsulated islets coated with AD-MSCs

pFUS treatment caused BG values to significantly decrease compared to the non-pFUS-treated group (i.e., emf-Islets vs. em-Islets) at days 4, 9, and 14 post-transplant (190 ± 28 vs. 260 ± 18, 207 ± 45 vs. 369 ± 50, and 196 ± 39 vs. 347 ± 67, respectively, *P* < 0.05). For pFUS-treated mice, BG values were not significantly different compared to their own baseline (i.e., their pre-transplantation values) throughout the course of our study from days 1 to 30 except at days 1–7 and 11–12 (*P* < 0.05). When compared with other tested groups, BG values for mice transplanted with emf-Islets were significantly lower than mice transplanted with m-Islets except at days 1–3, 5–7, 10–14, and 20–21, e-Islets except at day 1, and islets only except at day 1 and 5–6 (*P* < 0.05; Fig. 4t). At week 2, a significantly higher normoglycemia% was achieved for pFUS-treated mice compared to mice transplanted with Islets or e-Islets or m-Islets. However, from weeks 3 to 4, this effect was higher for pFUS-treated mice compared to all other tested groups (*P* < 0.05; Fig. 4u). Following IPGTT testing, mice transplanted with emf-Islets showed a significant decrease in BG values at 30 min compared to mice transplanted with em-Islets (*P* < 0.05). From 30 to 120 min, mice transplanted with emf-Islets showed a significant decrease in BG values compared to mice transplanted with m-Islets or e-Islets or Islets (*P* < 0.05; Fig. 4v). The AUC_0-120min_ of mice transplanted with emf-Islets was significantly lower compared to other tested groups (i.e., mice transplanted with em-Islets or m-Islets or e-Islets or Islets, *P* < 0.05; Fig. 4w). Furthermore, mice transplanted with emf-Islets showed a significantly enhanced BG clearance rate compared to mice transplanted with em-Islets or m-Islets or Islets (*P* < 0.05; Fig. 4x). Following pFUS treatment, the body weight of transplanted mice with em-Islets significantly increased (*P* < 0.05). When compared to all other tested groups, the body weight of mice in the pFUS-treated group was significantly higher at weeks 3 (*P* < 0.05) and 4 (*P* < 0.05; Fig. 4y).

#### Histological and molecular analyses

**Fig. 5:**
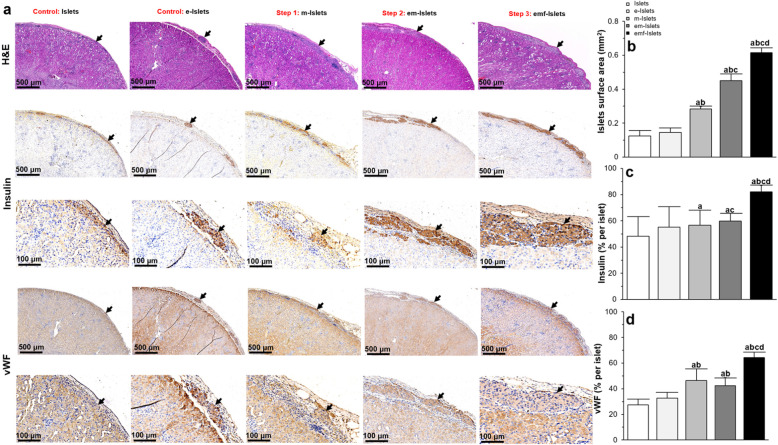
In vivo analysis of islet survival and function (histological analysis): **a** representative images following H&E, insulin, and vWF immunohistochemical staining of islets in tested groups (i.e., Islets, e-Islets, m-Islets, em-Islets, and emf-Islets) that were transplanted under the kidney capsule. Black arrows indicate islets present within the representative images and red arrows indicate blood vessels within the islets; **b** quantification of surface area occupied by islets; **c**–**e** quantification of positive **c** insulin and **d** vWF staining. Results were analyzed with at least 15–20 islets from 5 different sections through the kidney of each animal. Significant differences: ^a^*P* < 0.05: Islets vs. e-Islets or m-Islets or em-Islets or emf-Islets; ^b^*P* < 0.05: e-Islets vs. m-Islets or em-Islets or emf-Islets; ^c^*P* < 0.05: m-Islets vs. em-Islets or emf-Islets; ^d^*P* < 0.05: em-Islets vs. emf-Islets (one-way ANOVA post hoc Tukey test)

When tissues sections were stained using hematoxylin and eosin (H&E), and insulin, we found that encapsulation did not affect islet morphology (Fig. 5a) or islet number associated with the islet total surface area (0.13 ± 0.03 vs. 0.15 ± 0.03 mm^2^, *P* > 0.05; Fig. 5b). Results showed that there was no significant difference in insulin (48.27 ± 14.99 vs. 55.05 ± 15.77%/islet, *P* > 0.05; Fig. 5c) and vWF (32.57 ± 4.61 vs. 27.33 ± 4.68% per islet, *P* > 0.05; Fig. 5d) expressions levels within islets, regardless of whether or not they were encapsulated.

##### Step 1: AD-MSC coating on islets

In contrast to transplanted islets alone or e-Islets which lost their spherical morphology and demonstrated a more disorganized architecture, m-Islets were more spherical (Fig. 5a) with a significantly higher total surface area compared to islets (0.28 ± 0.20 vs. 0.13 ± 0.03 mm^2^, *P* < 0.05) or e-Islets (0.28 ± 0.20 vs. 0.15 ± 0.03 mm^2^, *P* < 0.05; Fig. 5b). When transplanted islets are healthier, they are normally intact and have a spherical structure [34–36]. However, when islets start to die, they lose their shape due to a loss in plasma membrane integrity and cell death [37]. In our previous study, we examined the use of AD-MSCs to help facilitate islet engraftment [38]. Here, our results support the cytoprotective effect provided by AD-MSCs, given that islet survival and function was improved when AD-MSCs were coated on islets; accordingly, m-Islets had a more spherical and organized structure when compared to transplanted islets alone. m-Islets showed a significant increase in insulin expression compared to Islets (56.54 ± 11.66 vs. 48.27 ± 14.99, *P* < 0.05; Fig. 5c). m-Islets also showed a significant increase in vWF expression compared to Islets (46.38 ± 9.11 vs. 27.33 ± 4.68% per islet, *P* < 0.05) or e-Islets (46.38 ± 9.11 vs. 32.57 ± 4.61% per islet, *P* < 0.05; Fig. 5d).

##### Step 2: Encapsulation of islets coated with AD-MSCs

Encapsulation of m-Islets resulted in islets retaining their spherical morphology (Fig. 5a) and enhancing their total surface area compared to Islets (0.45 ± 0.04 vs. 0.13 ± 0.03 mm^2^, *P* < 0.05), e-Islets (0.45 ± 0.04 vs. 0.15 ± 0.03 mm^2^, *P* < 0.05), and m-Islets (0.45 ± 0.04 vs. 0.28 ± 0.20 mm^2^, *P* < 0.05; Fig. 5b). In em-Islets group, islets also showed elevated insulin expression compared to Islets (59.82 ± 5.79 vs. 48.27 ± 14.99%/islet, *P* < 0.05) or m-Islets (59.82 ± 5.79 vs. 56.54 ± 11.66%/islet, *P* < 0.05; Fig. 5c). em-Islets also showed a significant increase in vWF expression as compared to Islets (42.51 ± 6.13 vs. 27.33 ± 4.68% per islet, *P* < 0.05) or e-Islets (42.51 ± 6.13 vs. 32.57 ± 4.61% per islet, *P* < 0.05) but no difference compared to m-Islets (42.51 ± 6.13 vs. 46.38 ± 9.11% per islet, *P* < 0.05; Fig. 5d).

##### Step 3: pFUS treatment on encapsulated islets coated with AD-MSCs

Following pFUS treatment, islets had retained their native size and spherical morphology and maintained their intrinsic architecture with β cells (positive insulin staining) located in the center of the islets. Of note, islets which were treated with pFUS also subjectively demonstrated vascular regions within islets (Fig. 5a, b). However, compared to other experimental groups (i.e., Islets, e-Islets, m-Islets, and em-Islets), pFUS treatment caused transplanted islets to show a significantly enhanced total surface area (0.61 ± 0.03 vs. 0.13 ± 0.03, 0.28 ± 0.2, and 0.45 ± 0.04 mm^2^, *P* < 0.05; Fig. 5b), insulin (82.22 ± 4.91 vs. 48.26 ± 14.99, 55.05 ± 15.77, 56.53 ± 11.66 and 59.82 ± 5.78%/islet, *P* < 0.05; Fig. 5c), and vWF (64.38 ± 4.16 vs. 27.33 ± 4.68, 32.57 ± 4.61, and 46.37 ± 9.11 and 42.51 ± 6.13%/islet, *P* < 0.05; Fig. 5d) expression.

**Fig. 6:**
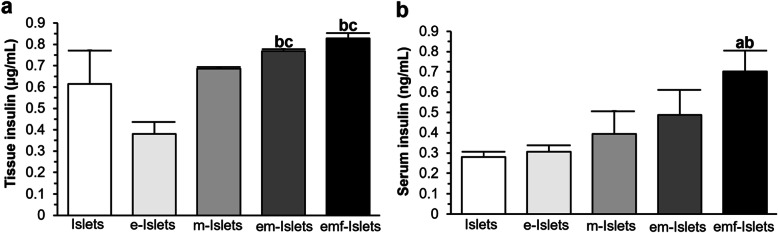
In vivo analysis of islet survival and function (molecular analysis): the level of insulin within **a** the kidney and **b** blood serum of mice transplanted with islets (measured with insulin ELISA). The **c** cytokine expression profile in the kidney of mice transplanted with islets (measured with multiplex ELISA) in following tested groups: Islets, e-Islets, m-Islets, em-Islets, and emf-Islets. Significant differences: ^a^*P* < 0.05: Islets vs. e-Islets or m-Islets or em-Islets or emf-Islets; ^b^*P* < 0.05: e-Islets vs. m-Islets or em-Islets or emf-Islets; ^c^*P* < 0.05: m-Islets vs. em-Islets or emf-Islets; ^d^*P* < 0.05: em-Islets vs. emf-Islets (one-way ANOVA post hoc Tukey test)

Analysis of the explanted kidneys showed that mice kidneys transplanted with emf-Islets contained a significantly higher amount of insulin compared to mice kidneys transplanted with e-Islets (0.83 ± 0.03 vs. 0.38 ± 0.06 μg/mL, *P* < 0.05) or m-Islets (0.83 ± 0.03 vs. 0.69 ± 0.01 μg/mL, *P* < 0.05; Fig. 6a). Similarly, a significantly increase in blood serum insulin was found for mice transplanted with emf-Islets compared to mice transplanted with Islets (0.70 ± 0.10 vs. 0.28 ± 0.03 ng/mL, *P* < 0.05) or e-Islets (0.70 ± 0.10 vs. 0.30 ± 0.03 ng/mL, *P* < 0.05; Fig. 6b).

**Fig. 7:**
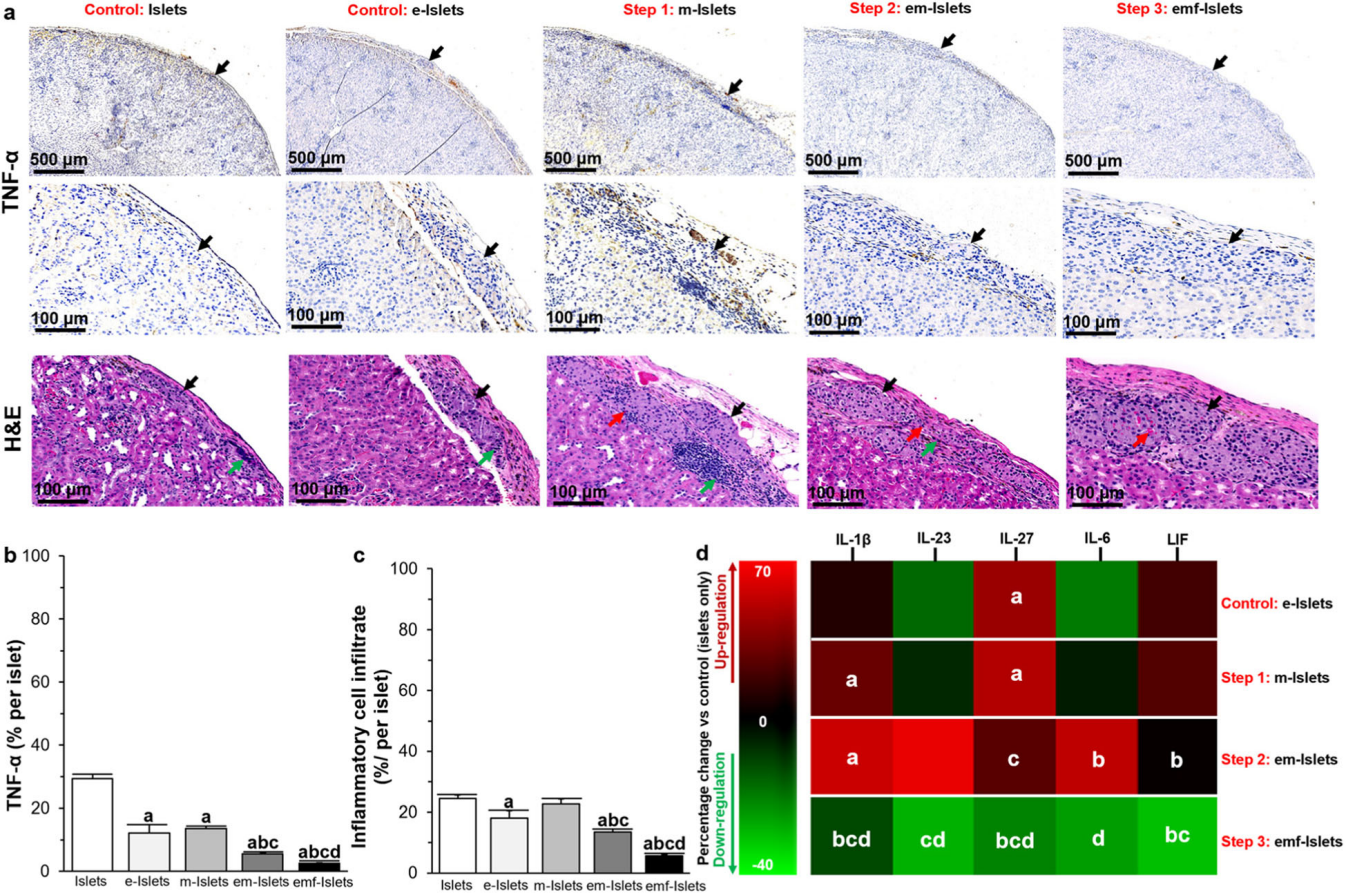
**a** Representative images following TNF-α immunohistochemical staining and H&E staining of islets that were transplanted under the kidney capsule. Black arrows indicate islets present within the representative images; quantification of **b** positive TNF-α staining, **c** inflammatory cell infiltrate; and **d** the cytokine expression profile in the kidney of mice transplanted with islets (measured with multiplex ELISA) in following tested groups: Islets, e-Islets, m-Islets, em-Islets, and emf-Islets. Significant differences: **b**–**d** ^a^*P* < 0.05: Islets vs. e-Islets or m-Islets or em-Islets or emf-Islets; ^b^*P* < 0.05: e-Islets vs. m-Islets or em-Islets or emf-Islets; ^c^*P* < 0.05: m-Islets vs. em-Islets or emf-Islets; ^d^*P* < 0.05: em-Islets vs. emf-Islets (one-way ANOVA post hoc Tukey test)

The expression of TNF-α was significantly reduced following encapsulation (29.33 ± 1.48 vs. 12.16 ± 2.65% per islet for Islets vs. e-Islets, *P* < 0.05). Decreased TNF-α expression was also noted for m-Islets compared to Islets (13.60 ± 0.77 vs. 29.33 ± 1.48% per islet, *P* < 0.05). When TNF-α expression of em-Islets was compared with other tested groups, it showed a significant decrease compared to Islets (5.55 ± 0.24 vs. 29.33 ± 1.48% per islet, *P* < 0.05), e-Islets (5.55 ± 0.24 vs. 12.16 ± 2.65% per islet, *P* < 0.05), and m-Islets (5.55 ± 0.24 vs. 13.60 ± 0.77% per islet, *P* < 0.05). Furthermore, in pFUS-treated mice, islets had less inflammation as demonstrated by a reduction of TNF-α staining when compared to other mice (2.65 ± 0.09 vs. 29.33 ± 1.48, 12.15 ± 2.65, 13.60 ± 0.77 and 5.55 ± 0.24% per islet, *P* < 0.05; Fig. 7a, b). In addition, there was also a corresponding reduction in the amount of inflammatory cell infiltrate for emf-Islets when compared with other tested groups, such as em-Islets, m-Islets, e-Islets, and Islets (5.65 ± 0.54 vs. 13.55 ± 0.97, 22.60 ± 1.77, 18.15 ± 2.41, and 24.41 ± 1.21% per islet *P* < 0.05; Fig. 7a, c).

We also analyzed the cytokine expression in kidneys which had received islet transplants and found that when pFUS is applied to em-Islets, pro-inflammatory cytokines were downregulated compared transplants of either Islets, e-Islets, m-Islets, or em-Islets. These cytokines were IL-1β (− 11.49 ± 0.70 vs. 10.01 ± 0.54, 29.32 ± 2.20, 55.86 ± 7.73%, *P* < 0.05), IL-23 (− 27.89 ± 1.23 vs. 6.17 ± 0.25, and 64.28 ± 13.98%, *P* < 0.05), IL-27 (− 20.62 ± 2.49 vs. 42.98 ± 2.67, 49.28 ± 2.47, and 24.50 ± 1.75%, *P* < 0.05), IL-6 (− 24.65 ± 1.06 vs. 52.44 ± 9.61%, *P* < 0.05), and leukemia inhibitory factor (LIF: − 29.98 ± 7.03 vs. 18.88 ± 0.41 and 23.13 ± 2.91%, *P* < 0.05; Fig. 7d).

## Discussion

In the present study, we demonstrated that we can promote the function and engraftment of pancreatic islets using a novel three-step approach. Here, we combined a cellular therapy shown to promote islet function (i.e., using AD-MSCs to coat Islets; step 1) with a biocompatible biomaterial shown to protect transplanted islets (i.e., using alginate to encapsulate m-Islets; step 2) and then we used a novel non-invasive technology, which employs sound waves, to stimulate both islets and AD-MSCs (i.e., applying pFUS to em-Islets; step 3). In step 1, we co-cultured islets and AD-MSCs in a 1:500 ratio for 24 h to enable AD-MSCs to attach and uniformly coat Islets. In step 2, m-Islets were conformally encapsulated with an alginate layer measuring 50 ± 10 μm to spatially localize AD-MSCs to islets as well as to provide a protective barrier to islets from any immune mediated attack. Finally, in step 3, em-Islets were treated with pFUS using the following parameters: 100 Hz PRF, 20% DC, 16.5 Vpk-pk, and 1 min exposure time. This was done to enable sound waves to increase the function and survival of islets either directly or indirectly by stimulating AD-MSCs associated with the islets. Our in vitro and in vivo data both demonstrated an additive and synergistic effect on islet function and survival when these 3 steps were employed together.

Clinical studies have shown that islet transplantation can provide diabetic patients with long-term insulin independence and normalization of glycosylated hemoglobin (HbA1c) levels, while also preventing hypoglycemic episodes [39–42]. However, despite more than 80% of patients becoming insulin-independent within the first year following islet transplantation, this number reduces over 5 years [43]. Hence, islet transplantation is yet to reach its full clinical potential which, in part, can be attributed to islets being lost either immediately following their transplantation (i.e., failure of engraftment) or later on (i.e., as a result of autoimmune mediated cellular rejection). To address these shortcomings, in previous studies, we and others have examined the use of MSCs to help facilitate islet engraftment [38] as well as encapsulation to reduce the need for stringent immunosuppression to prevent graft rejection [44]. However, by combining both of these approaches, this could potentially provide a complementary strategy to simultaneously address both the above issues.

Over the past decade, MSCs from different sources have been studied with islets*.* In in vitro studies where islets and MSCs are co-cultured together, and hence spatially constrained within a defined environment, MSCs have been shown to increase islet survival and function in both normal and adverse conditions [11, 45–47]. These beneficial effects of MSCs have also been seen when translated in vivo when islets and MSCs are co-transplanted together in a confined environment, such as the kidney subcapsular space [38]. Here, MSCs have been shown to improve islet revascularization as well as suppress inflammatory responses [48, 49]. However, during clinical islet transplantation, islets are infused into the portal vein, which results in them being randomly distributed throughout the liver. When MSCs are then also administered, they are given in another separate infusion that does not ensure co-engraftment of both the islets and MSCs at the same location. Furthermore, the much smaller MSCs (15–30 μm) can actually pass through the liver with most cells eventually ending up in the lung microcirculation [50, 51]. If MSCs cannot be spatially located next to the islets, this will limit their therapeutic effect which is predominantly based on their ability to sense and modulate their surrounding microenvironment via their paracrine action [52]. Given that MSCs, and in particular AD-MSCs, have such a prominent beneficial effect on islets, we decided to not only coat islets with these cells, but to also encapsulate them together to prevent them from dissociating at the time of transplantation.

In a previous work, Duprez et al. [25] demonstrated that human bone marrow MSCs surrounded human islets before migrating towards their center and that the MSC coating was both dose- and time-dependent. They used islets and MSCs with the ratio of 1:100–500 and made comparisons between MSC coated islets; their data showed that with ratios of 1:100 (islet: MSCs), there was only a sporadic binding of MSCs to the islet surface; however, when the ratio increased to > 100 MSCs, this resulted in a more uniform MSC coating. Indeed, this group found that a ratio of 1:500 islet: MSCs, and a coating time of 24 h, resulted in the optimal and uniform coating of islets with MSCs [25], and this was also verified by our studies using AD-MSCs. Once islets are coated with AD-MSCs, we found they exhibited an enhanced secretion of insulin in response to glucose challenges as well as improved survival in vitro which we attributed to the ability of AD-MSCs to secrete trophic and growth factors [10, 46] as well as increase the insulin sensitivity of islets [53].

In order to keep AD-MSCs together with islets, we conformally encapsulated islets coated with AD-MSCs in a thin layer of alginate (50 ± 10 μm). While non-conformal encapsulation of islets have been extensively studied (i.e., using 500 μm capsules), these are not clinically translatable using the current approach for islet transplantation given that it increases the average diameter of conventional islet by approximately three fold which then results in an increase in the transplant volume by approximately 27 times, which can be difficult to accommodate in the host’s liver [54, 55]. Furthermore, this type of encapsulation predisposes islets to developing hypoxia given that the diffusion distance of oxygen through such thick capsules is hindered [54]. Encapsulation also prevents the revascularization process which further exacerbates the hypoxia situation and also hinders the release of insulin compared to non-encapsulated islets [56]. Hence, recent studies have been examining encapsulation techniques in which a very thin membrane, or conformal coating, can be applied to islets. By using conformal coating to minimize capsule thickness, this will help islets to better engraft in small spaces (i.e., the hepatic sinusoids) [57]. Conformal coating can also help sustain islet function by facilitating the rapid diffusion of oxygen and nutrients through the thin coating, as well as the release of insulin from islets in response to glucose [58]. Hence, in the present work, we used an air flow technique which enabled us to uniformly coat islets with a 50 ± 10 μm layer of alginate which we confirmed with confocal microscopy. At this thickness, the alginate layer still allows for the diffusion of oxygen, nutrients, and glucose to islets while concomitantly protecting them from immune attack [59]. Moreover, the alginate layer can prevent islet aggregation and preserve islet morphology [60] both of which have been shown to improve islet function [61]. We chose alginate as our biomaterial for encapsulation given that it is one of the most widely investigated cell encapsulation biomaterials [62] and has been used in several clinical trials [63–66]. Our results confirm that that islet survival and function can be improved in vitro following encapsulation and encapsulated islets retained their islet-like morphology in vivo. We also found a significant improvement in islet function and engraftment with encapsulated islets coated with AD-MSCs compared to when islets alone were encapsulated. This effect can be potentially due to the continuous exposure of the islets to the AD-MSCs as a result of encapsulation, which may confer an advantage for the lifetime of the graft. Furthermore, alginate is inherently non-degradable in vivo due to the lack of the required enzyme to cleave the polymer chains [67]. This is the main reason why alginate polymers have been extensively used in islet transplantation [68–70]. Research has also shown that the degradation of alginate is a function of pH (i.e., alginate is stable in acidic pH but swells and dissolves in alkaline pH [71]). Following the surgery, the pH of the site of islet transplantation is expected to be low [72] due to post-surgical inflammation [73]. Hence, our thin 50 ± 10 μm layer of alginate around m-Islets should remain stable following transplantation.

One question that still needs to be addressed is how to stimulate islets, as well as other cellular therapies like AD-MSCs, after they have been given into living subjects. One approach to non-invasively stimulate these cells is to use sound waves. The ability of sound waves to propagate through tissue, and be focused at specific locations deep within the body, makes pFUS a very appealing non-invasive therapeutic strategy. Our previous research has shown that pFUS is able to enhance the ability of islets to secrete insulin via a calcium dependent mechanism [19]. Furthermore, given the ability of MSCs to be stimulated by their surrounding environment (i.e., hypoxia [74]), temperature (i.e., thermal shock [75]), and even chemicals (i.e., pharmacologic treatment or pro-inflammatory cytokine exposure [76, 77]), it is not surprising that sound waves, at specific intensities, can physically stimulate AD-MSCs via a biomechanical effect [78]. Stimulated MSCs have also been shown to upregulate Toll-like receptors (TLRs), which can increase their function to inflammatory milieu [79]. Although future work will examine the specific mechanisms by which pFUS stimulates MSCs, we found that when encapsulated islets coated with AD-MSCs were stimulated with pFUS there was improved islet survival (i.e., enhanced percentage of live cells) and function (i.e., enhanced glucose-stimulated insulin secretion). Although, we did not track MSCs survival in this study, future studies will label MSCs and explore the effect of encapsulation and pFUS treatment on MSC survival in vivo.

Based on our in vitro data, we then examined whether this approach could be translated into an animal model. Hence, in diabetic animals, we transplanted alginate encapsulated islets that had been coated with AD-MSCs and then used pFUS to stimulate these cells over 2 weeks (i.e., over the period of islet engraftment and when most islets are lost as a result of hypoxia, nutrient deprivation and inflammation). Interestingly, we found that by using this combined three-step approach, we were able to restore glycemic control in animals faster and with less variability. These animals were also able to respond quicker and faster to intraperitoneal glucose challenges with transplanted islets also demonstrating reduced evidence of surrounding inflammation (shown by a decreased expression of TNF-α on histology as well as downregulation of pro-inflammatory cytokines IL-1β [80], IL-23 [81], IL-27 [82], and IL-6 [83] in the tissue lysate of the islet transplant). Of the pro-inflammatory cytokines which were down regulated, IL-1β is key given the upregulation of this specific one has been shown to be deleterious to transplanted islet survival and function via stimulation of insulin resistance in islets [84], inhibition of beta cell function [85], promotion of Fas-triggered apoptosis [85], and induction of nitric oxide (NO) synthase in beta cells and subsequent generation of toxic NO levels [86]. The representative vWF and H&E images also confirm an enhanced vascularization following AD-MSC coating and pFUS treatment on islets.

Hence, taken together, these results demonstrate the ability of this approach to not only help islet engraftment at the site of transplantation but also that promote islet survival and function. Given our in vitro data, it is likely that pFUS is working to both stimulate islets directly as well as indirectly through the stimulation of AD-MSCs which are coated onto the surface of islets. In addition, the alginate capsule will likely also protect the transplanted islets from direct effects of inflammation as well as any host-mediated response [87]. In future, additional studies will examine apoptosis and proliferation within transplanted islets using TUNEL and Ki67 assays.

The clinical translation of this approach for patients with T1D treated with islet transplantation is feasible given that pFUS can be applied to patients using current clinically available equipment and AD-MSCs have already been used in multiple clinical trials (NCT03265613, NCT03691909, NCT02407470, NCT02145897). Although the acoustic parameters of pFUS to achieve the PNPs and intensities reported here would need to be modified accordingly, it should be noted that these values measured here are non-derated values given that the coupling medium (water) is non-attenuating, and the depth at which the pFUS was applied in the animals was non-significant for the frequency utilized. For humans, it will be necessary to utilize acoustic parameters that achieve the reported PNPs and intensities after deration. Derating the PNPs and intensities will be necessary because, for clinical treatment, the transducer will be coupled directly to the individual (via an acoustic coupling gel) and the acoustic pressure and intensities will be attenuated by the intervening tissue between the transducer and the target tissue region. Of note, clinical trials have also been carried out using encapsulated islets [65, 66], though none of these were able to achieve insulin independence [88]. Clinical trials using encapsulated islets have lacked long-term efficacy and, although generally considered clinically safe, have not been encouraging overall [88]. However, considering that clinical trials conducted with encapsulated islets were shown to be safe [66], we believe a similar evaluation in patients with our three-step approach using AD-MSCs, encapsulation, and pFUS may provide therapeutic benefit.

## Conclusion

In summary, we have demonstrated the beneficial effect of a three-step approach for islet transplantation: islets coated with AD-MSCs, alginate encapsulation, and pFUS treatment. We have shown that our approach improves the overall survival and function of transplanted islets with a corresponding increase in angiogenesis and reduction in inflammation. Hence, this approach may overcome many of the hurdles currently faced by islet transplantation that have thus far limited it from reaching its full clinical potential.

## Supplementary information

**Additional file 1.**

## Data Availability

All data discussed in the paper will be made available to readers upon request.

## Abbreviations

T1D: Type 1 diabetes
AD-MSCs: Adipose tissue-derived mesenchymal stem cells
FUS: Focused ultrasound
pFUS: Pulsed focused ultrasound
PRF: Pulse repetition frequency
DC: Duty cycle
PNP: Peak negative pressure
I_sptp_: Spatial average pulse average intensity
IBMIR: Instant blood-mediated inflammatory reaction
*V*_*m*_: Membrane potential
IACUC: Institutional Animal Care and Use Committee
HEPES: 4-(2-Hydroxyethyl)-1-piperazineethanesulfonic acid
Ca: Calcium
C: Carbon
O: Oxygen
GSIS: Glucose-stimulated insulin secretion
BG: Blood glucose
IPGTT: Intraperitoneal glucose tolerance tests
H&E: Hematoxylin and eosin
IL-1β: Interleukin-1beta
IFN-γ: Interferon-gamma
TNF-α: Tumor necrosis factor-alpha
IL-23: Interleukin-23
IL-27: Interleukin-27
IL-6: Interleukin-6
LIF: Leukemia inhibitory factor
NO: Nitric oxide
SEM: Standard error of the mean
ANOVA: Analysis of variance
