# Correction: Facilitating islet transplantation using a three-step approach with mesenchymal stem cells, encapsulation, and pulsed focused ultrasound

**DOI:** 10.1186/s13287-022-03210-6

**Published:** 2022-12-20

**Authors:** Mehdi Razavi, Tanchen Ren, Fengyang Zheng, Arsenii Telichko, Jing Wang, Jeremy J. Dahl, Utkan Demirci, Avnesh S. Thakor

**Affiliations:** 1 Department of Radiology, Interventional Regenerative Medicine and Imaging Laboratory, Stanford University School of Medicine, 3155 Porter Drive, Palo Alto, CA 94304 USA; 2 Biionix™ (Bionic Materials, Implants & Interfaces) Cluster, Department of Internal Medicine, College of Medicine, University of Central Florida, Orlando, FL 32827 USA; 3 Department of Materials Science and Engineering, University of Central Florida, Orlando, FL 32816 USA; 4 Department of Radiology, Bio-Acoustic MEMS in Medicine Laboratory (BAMM), Stanford University School of Medicine, Palo Alto, CA 94304 USA; 5 Department of Radiology, Dahl Ultrasound Laboratory, Stanford University School of Medicine, Palo Alto, CA 94304 USA

**Correction: Stem Cell Research & Therapy (2020) 11:405** **https://doi.org/10.1186/s13287-020-01897-z**

In the original article, the authors identified an editing error in Fig. 3a where the Live image used in Step 2: em-islets INF-Y was incorrect from a mistake during figure assembly. The correct Fig. 3 has now been provided here and the authors apologize for any inconvenience caused.

**Fig. 3:**
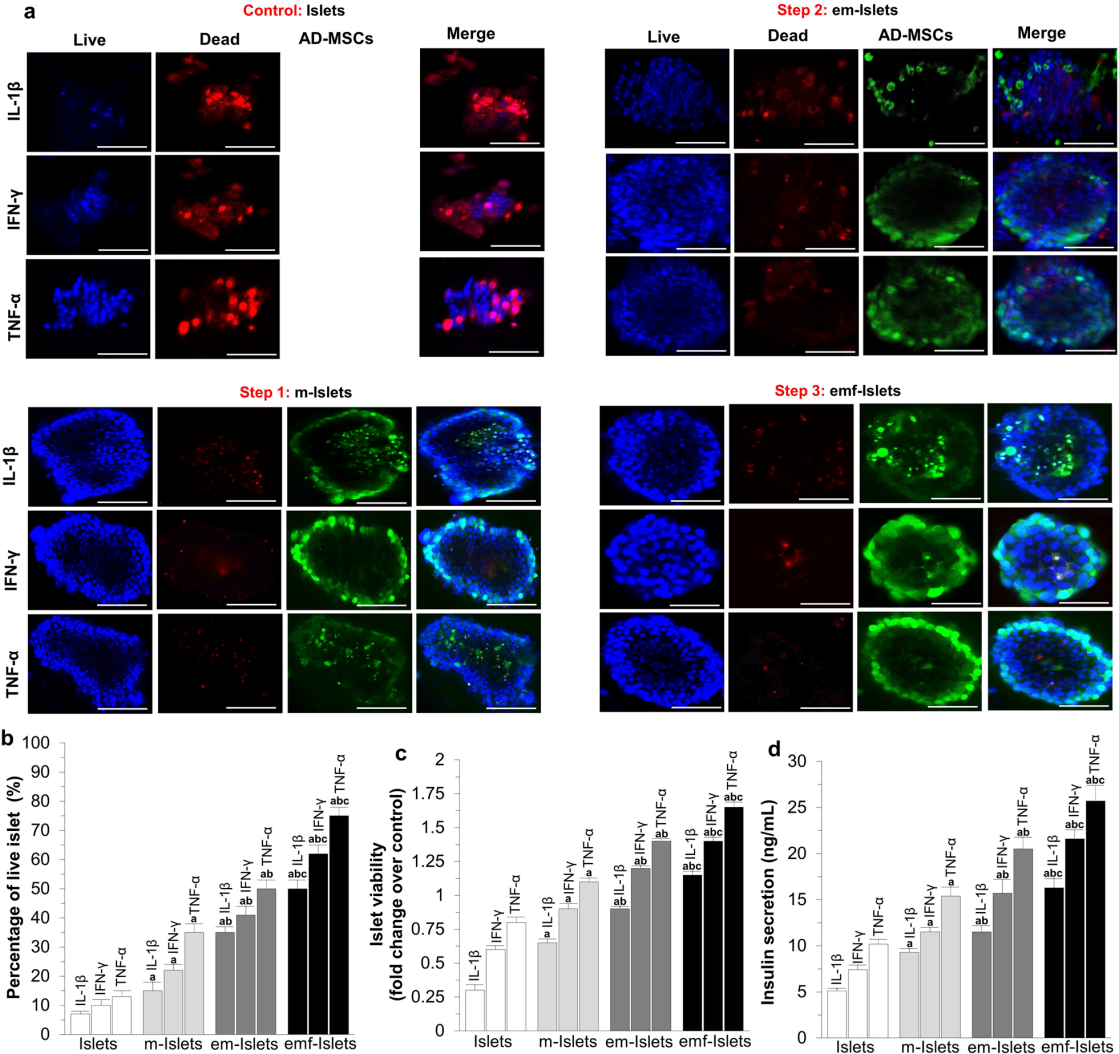
In vitro analysis of islet survival and function following exposure to pro-inflammatory cytokines: **a** representative confocal images and results of **b** live/dead, **c** MTT, and **d** high-glucose-stimulated insulin secretion assays of tested groups (i.e., Islets, e-Islets, m-Islets, em-Islets, and emf-Islets). Blue: live cells stained with Hoechst. Red: dead cells stained with PI. Green: AD-MSCs stained with FDA. Scale bar = 50 μm. Significant differences: **b–d** ^a^*P* < 0.05: Islets vs. m-Islets or em-Islets or emf-Islets; ^b^*P* < 0.05: m-Islets vs. em-Islets or emf-Islets; ^d^*P* < 0.05: em-Islets vs. emf-Islets (one-way ANOVA post hoc Tukey test)

