# Supplementary material for: Facilitating islet transplantation using a three-step approach with mesenchymal stem cells, encapsulation, and pulsed focused ultrasound
Source: Stem Cell Res Ther. 2020 Sep 18;11:405. doi: 10.1186/s13287-020-01897-z (PMC7501701; doi:10.1186/s13287-020-01897-z)
Supplement: Supplementary file 1 — Additional file 1. [file 13287_2020_1897_MOESM1_ESM.docx]

**SUPPLEMENTAL INFORMATION**

**FACILITATING ISLET TRANSPLANTATION USING A THREE-STEP APPROACH WITH**

**MESCENCHYMAL STEM CELLS, ENCAPSULATION, AND PULSED FOCUSED ULTRASOUND**

**MEHDI RAZAVI^1,2,3^, TANCHEN REN^3^, FENGYANG ZHENG^1^, ARSENII TELICHKO^4^, JING WANG^1^, JEREMY DAHL^4^, UTKAN DEMIRCI^3^ & ^*^AVNESH S THAKOR^1^**

^1^Interventional Regenerative Medicine and Imaging Laboratory, Stanford University School of Medicine, 3155 Porter Drive, Department of Radiology, Palo Alto, California 94304, USA

^2^Biionix^TM^ (Bionic Materials, Implants & Interfaces) Cluster, Department of Internal Medicine,

College of Medicine, University of Central Florida, Orlando, Florida 32827, USA

^3^Department of Materials Science and Engineering, University of Central Florida,

Orlando, FL 32816, USA

^3^Bio-Acoustic MEMS in Medicine Laboratory (BAMM), Stanford University School of Medicine, Department of Radiology, Palo Alto, California 94304, USA

^4^Dahl Ultrasound Laboratory, Stanford University School of Medicine, Department of Radiology, Palo Alto, California 94304, USA

**Journal:** *Stem Cell Research & Therapy*

**Article Type:** Research Paper

**Key Words:** Islets Transplantation; Mesenchymal Stem Cells; Encapsulation; Pulsed Focused Ultrasound; Diabetes.

**Corresponding Authors:** Avnesh S Thakor, MD PhD

E-mail: [asthakor@stanford.edu](mailto:asthakor@stanford.edu)

Utkan Demirci PhD

E-mail: [utkan@stanford.edu](mailto:asthakor@stanford.edu)

**MATERIALS AND METHODS**

**1. Islet and AD-MSCs Isolation and Characterization**

**1.1. Islet Isolation**

Pancreatic islets were isolated from C57/B6 mice (male, 6-8 week-old, Charles River Laboratories, USA), as previously described (1). In brief, the common bile duct was cannulated with a 30G needle and the pancreas distended with 3ml of cold collagenase solution (Fischer Scientific, USA). The pancreas was then removed and islets isolated by digesting the pancreas at 37°C for 10min followed by purification using histopaque-density gradients. Islets were then washed with Hank's balanced salt solution (HBSS, Gibco, USA) supplemented with 0.1% bovine serum albumin (BSA, Gibco, USA). Finally, islets were cultured in a humidified incubator at 37°C and 5% CO_2_ with Roswell Park Memorial Institute medium (RPMI; Gibco, USA) supplemented with 10% fetal bovine serum (FBS; Invitrogen, USA) and 50U/mL penicillin-50µg/mL streptomycin.

**1.2. AD-MSCs Isolation and Characterization**

Mouse adipose tissue was obtained from the lower abdomen in male C57BL/6 mice at 6-8 weeks of age, as previously described (2). In brief, the procured adipose tissue was washed with sterile phosphate buffered saline (PBS), minced with scissors and then digested with 1 mg/mL type I collagenase (Sigma-Aldrich) in serum-free medium at 37^o^C for 3hr. The digestion was then inactivated with an equal volume of DMEM (Gibco) supplemented with 10% fetal bovine serum (FBS; Invitrogen). All samples were then filtered through a 100µm mesh filter to remove any debris. The cellular pellets were collected and then re-suspended in DMEM containing 10% FBS in a humidified incubator at 37^o^C with 5% CO_2_. Spindle-shaped cells appeared on Day 3, with cells reaching 70–80% confluence by 4-5 days; at this point, cells were then split and sub-cultured.

AD-MSCs from passage number 3-5 were used for all transplantation studies with cells examined using a Zeiss LSM710 Confocal Microscope. For cell surface marker expression, adherent AD-MSCs were detached, disaggregated into single cells, and then stained with the following antibodies for 40min at 4^o^C: Phycoerythrin (PE) conjugated mouse monoclonal antibodies against CD90, CD105 and Allophycocyanin (APC) conjugated mouse monoclonal antibody against CD45 (Biolegend). Following incubation, AD-MSCs were washed twice with PBS before being re-suspended with 0.5mL PBS at which point their surface marker expression compared to unstained AD-MSCs (as control) was determined using the Guava® easyCyte system (Millipore, Darmstadt, Germany).

**1.3. Encapsulation of Islets Coated with AD-MSCs**

The structure and chemistry of the alginate capsules were characterized using scanning electron microscopy (SEM, XL30 Sirion, FEI, USA) and X-ray photoelectron spectroscopy (XPS, ULVAC-PHI, Physical Electronics, USA), respectively. Alginate capsules were dehydrated using 10min sequential immersions through a standard sequence of 50, 70, 90 and 100% ethanol solutions. They were then coated with Au-Pd using a sputter coater and their morphology analyzed using SEM. Both qualitative and quantitative information about the presence of different elements on the surface of the alginate hydrogel were obtained using survey and high-resolution scans with a VersaProbe 1 Scanning XPS Microprobe and a monochromatic Al K alpha X-ray source. The survey scan was performed with a pass energy of 117.4eV, a range of 0-1400 eV, an energy step of 1 eV, a time/step of 20 ms and a total of 3 cycles. All spectra were collected with the charge neutralization flood gun turned on. All data was then processed using the MultiPak program XPS software package.

**1.4. pFUS Treatment on Encapsulated Islets Coated with AD-MSCs**

**1.4.1. In vitro**

*Set-up*

pFUS was performed on islets using a custom built system consisting of a function generator (33250A, Agilent, Santa Clara, CA), a power amplifier (ENI 525LA, Electronics & Innovation, Rochester, NY) and a focused piston transducer (2.54cm diameter, 5cm focal depth) operating at 1MHz center frequency.

*Output Characterization*

To measure the acoustic pressure and intensities for *in vitro* experiments, the transducer was submerged in a water tank containing degassed water and driven by sinusoidal ultrasound pulses at 1MHz frequency. The pulses were transmitted at a pulse repetition frequency (PRF) of 100Hz, 2000 cycles per pulse, and 20% duty cycle. A needle hydrophone (HNR0500, Onda Corporation, Sunnyvale, CA) was placed in front of the transducer and connected to an oscilloscope to measure the emitted acoustic waves. An Acoustic Intensity Measurement System (AIMS III, Onda Corporation., Sunnyvale, CA) was used to perform a raster scan of the pressure field with the hydrophone in the x-y plane 3 mm from the transducer surface. Digitized pressure waveforms were recorded with an oscilloscope (Agilent DSO6012a, Santa Clara, CA). A pressure field scan was performed for applied amplified voltage of 16.5 Vpk-pk. The peak negative pressure (PNP) and intensities for each pressure field scan were computed. The spatial-average temporal average intensity (I_sata_), spatial-average pulse-average intensity (I_sapa_), and the spatial-peak temporal-peak intensity (I_sptp_) were calculated. A second set of pressure field scans was performed by inserting a polystyrene 12-well plate between the hydrophone and transducer, with the transducer position underneath a single well filled with degassed water (2 mL). Only 5 cycles per pulse were utilized to minimize the interference of the acoustic pulses reflected from the well plate. The transducer was coupled to the plate using ultrasound gel (Aquasonic, Bio-Medical Instruments, USA). The hydrophone was submerged into the center of the well, 3mm away from the transducer, and pressure field scans were then obtained using the 3 voltages described previously.

**1.4.2. In Vivo**

*Set-up*

pFUS was administered to animals using a modified HIFU transducer (H-102NRE, Sonic Concepts, USA). This transducer has a 1.1MHz center frequency, a focal depth of 55mm, a 64mm outer diameter and a 49mm central opening to accommodate a diagnostic ultrasound imaging transducer. The transducer was driven by a function generator through a power amplifier, and had an impedance matching circuit (Sonic Concepts, USA). A diagnostic ultrasound transducer (14L5SP) connected to a Siemens ACUSON S2000 unit (Siemens Healthcare, Issaquah, WA) was utilized for guiding/targeting the pFUS treatment at the site of the islet transplantation.

*Calibration*

The HIFU transducer setup for acoustic output measurements was similar to the piston transducer calibration, except a fiber-optic hydrophone (FOH) (Precision Acoustics, Dorchester, UK) was used due to its ability to sustain high pressures induced by the HIFU transducer. The transducer was driven at 1.1MHz with 20 cycle bursts at a PRF of 100Hz (0.18% DC). The FOH hydrophone was placed at the focal spot of the transducer and the pressure fields and intensities were measured. The applied voltage was kept relatively low (measured PNP < 3 MPa) to eliminate the potential damage to the hydrophone. The measured beam width (FWHM) at the focal zone was 1.5mm in diameter and 10mm long. The measured PNPs and intensities were then scaled to the desired PRF (5 Hz) and DC (20%).

*Image Guidance*

For precise targeting, the imaging transducer was mechanically coupled to the HIFU transducer using a custom 3D-printed holder that enabled the focal spot of the ultrasound therapy beam to lie in the ultrasound imaging plane. The focal spot of the HIFU transducer was fixed at 55mm axial and 0mm lateral distance from the center point of the imaging transducer. The AIMS III system, hydrophone, oscilloscope and Siemens ACUSON S2000 unit were used to align the ultrasound beam and imaging plane to within a tolerance of 200μm. Mice that had received an islet transplant were first anesthetized and then inserted vertically, up to the level of their neck, into a water bath containing heated (37°C) degassed water to couple the ultrasound to the animal. The assembled holder with the HIFU transducer was then attached to a translation stage to enable translation of the focal spot and to keep it fixed during the pFUS therapy. Using real-time ultrasound imaging guidance, the mouse was positioned so the right kidney was in the center of the focal point of the HIFU transducer.

**1.5. In Vitro Islet Survival and Function**

Under a microscope and with a Gilson P 200 pipette, groups of 30 hand-picked, size-matched islets were suspended in the 200μl RPMI medium into 96-well plates, cultured for 7 days and islet survival and function were assessed during baseline/normal conditions or following exposure to pro-inflammatory cytokines. In the latter experiment, pro-inflammatory cytokine solutions (Biolegend, USA) consisting of 0.4625ng IL-1β or 1560ng IFN- γ or 250ng TNF-α (specific activities 1.16–0.5461, 2.4611, 5–26107 U/mg respectively) were made and collectively added to the culture medium at day 0. These cytokines were designed to represent the inflammatory cytokines islets are exposed to immediately following their transplantation (3-5). At day 7, islet survival and function were assessed.

**1.5.1. Islet Survival**

An MTT (4,5-dimethylthiazol-2-yl)-2,5-diphenyltetrazolium bromide assay was performed to determine islet viability as previously described (6). In addition, islets were examined using a Live/Dead assay, which used a staining solution consisting of Hoechst 33342 (for live cells; Thermofisher Scientific, USA), propidium iodide (PI; for dead cells, Thermofisher Scientific, USA) and fluorescein diacetate (FDA; for AD-MSCs labelling, Thermofisher Scientific, USA). The culture medium was carefully removed under a bright-field microscope, the staining solution (Hoechst 33342 (50µL/well), PI (75 µL/well) and FDA (75 µL/well)) was added and then left to incubate in darkness at 37°C/5% CO_2_. After 0.5h, the staining solution was removed and the live cell imaging solution (200µL/well, Thermofisher Scientific, USA) was added to each well before imaging. Imaging was conducted on cells into the uncoated 96-well glass plates (MatTek, USA) held at 37°C/5%CO_2_ on a Zeiss LSM710 Confocal Microscope with a 20× objective. Figures were created with the FIJI software (ImageJ, GNU General Public License) and the percentage of live to dead cells was estimated.

**1.5.2. Islet Function**

To assess islet function, a glucose stimulated insulin secretion (GSIS) assay was performed whereby islets were exposed to a Krebs-Ringer Buffer (KRB, Sigma Aldrich, USA) with a low glucose (basal: 2.8mM) for 2h followed by a KRB with high glucose (stimulus: 16.7mM) for 2h at 37°C/5% CO_2_. Supernatant samples were collected after each incubation period, frozen at -80°C and insulin levels quantified using a mouse insulin ELISA kit (Mercodia, USA).

**1.6. In Vivo Analysis of Islet Survival and Function**

**1.6.1. Metabolic analysis**

All metabolic analyses were performed in conscious, restrained mice at the indicated time points. For all tests, blood glucose was measured via tail vein sampling using a handheld glucometer (Bayer Contour Glucose Meter, USA). Mice were considered normoglycemic when non-fasting blood glucose levels were <200mg/dl (7). Intraperitoneal glucose tolerance tests (IPGTT) were performed at week 2 post-transplantation after overnight fasting and an injection of glucose (2g/kg). Blood glucose values were then measured at indicated time points allowing for the area under the curve (AUC) and blood glucose clearance rate to be calculated between transplantation groups.

**1.6.2. Histological and Molecular Analyses**

Mice were euthanized at day 30 post-transplant. At euthanasia, the kidneys containing the transplanted islets that had been treated with or without pFUS were harvested, and then proceed for either histological analysis (i.e. fixed in 4% paraformaldehyde (PFA), dehydrated with graded ethanol solutions, embedded in paraffin and sliced with a microtome) or for molecular analysis (i.e. tissues were stored at -80°C for subsequent processing).

Sections were prepared for histological and immunohistochemical analyses to determine islet structure and viability (Haemotoxylin and Eosin (H&E) and insulin staining), and evidence of inflammation (H&E and tumor necrosis factor alpha (TNF-α) staining) via standard procedures. The stained sections were then imaged using a NanoZoomer slide scanner 2.0-RS (Hamamatsu, Japan). Results were analyzed using FIJI Image J software with at least 15-20 islets from 5 different sections through the kidney of each animal.

At euthanasia, blood samples were also collected to measure the serum insulin levels (insulin ELISA kit; Mercodia). The frozen kidney tissue was then homogenized as follow: tissue samples were placed in a homogenization buffer at a ratio of 1 kidney/1mL buffer; the buffer contained a protease inhibitor combination (Sigma Aldrich, USA) including 4-(2-Aminoethyl)benzenesulfonyl fluoride hydrochloride (AEBSF, 2mM), Aprotinin (0.3μM), Bestatin (116μM), trans-Epoxysuccinyl-L-leucylamido(4-guanidino)butane (E-64, 14μM), Leupeptin (1μM) and ethylenediaminetetraacetic acid (EDTA, 1mM) in tissue protein extraction reagent (ThermoFisher Scientific, USA) containing phenylmethylsulfonyl fluoride (PMSF). All homogenized kidney samples were sonicated 3 times for a total of 8s (Branson SLPe) and then placed on a rotisserie at 4°C for 45min before being centrifuged at 4°C, 15000rpm for 15min. The tissue supernatant was then collected and the insulin content measured (mouse insulin ELISA kit; Mercodia) as well as the level of tissue cytokines (mouse multiplex ELISA; eBiosciences/Affymetrix/Fisher). In brief, beads were first added to a 96 well plate and washed (Biotek ELx405). Samples were then added to the plate containing the mixed antibody-linked beads and incubated at room temperature for 1h followed by overnight incubation at 4°C on a plate shaker (500rpm). Biotinylated detection antibody was then added, after which the plates were incubated at room temperature for 75min on the plate shaker (500rpm). Next, the samples were washed and streptavidin-PE added followed by incubation of the plate 30min at room temperature on the plate shaker (500rpm). The plate was then washed and a reading buffer added to all the wells. Finally, a Luminex Flex 3D instrument was used to read the plates with a lower bound of 50 beads per sample per cytokine. Control assay beads (Radix Biosolutions) were added to all wells. Multiplex ELISA assays were performed in 3 animals from each group. The average cytokine value was taken from 2 separate readings. The fold change in cytokine expression in the transplanted islets treated with pFUS vs. control transplanted islets (i.e. non-treated islets) was calculated using Eq. S1:

$$Foldchangevs.control=\frac{{OD}_{sample}-{OD}_{control}}{{OD}_{control}}$$

(**Eq. S1**)

${OD}_{sample}$: optical density (absorbance) of transplanted islets treated with pFUS; ${OD}_{control}$: optical density (absorbance) of control transplanted islets.

**References**

1. Neuman JC, Truchan NA, Joseph JW, & Kimple ME (2014) A method for mouse pancreatic islet isolation and intracellular cAMP determination. *Journal of visualized experiments: JoVE* (88).

2. Sung JH*, et al.* (2008) Isolation and characterization of mouse mesenchymal stem cells. *Transplant Proc* 40(8):2649-2654.

3. Yeung TY*, et al.* (2012) Human mesenchymal stem cells protect human islets from pro-inflammatory cytokines. *PloS one* 7(5):e38189.

4. Barshes NR, Wyllie S, & Goss JA (2005) Inflammation-mediated dysfunction and apoptosis in pancreatic islet transplantation: implications for intrahepatic grafts. *J Leukoc Biol* 77(5):587-597.

5. Matsuda T*, et al.* (2005) Inhibition of p38 pathway suppresses human islet production of pro-inflammatory cytokines and improves islet graft function. *Am J Transplant* 5(3):484-493.

6. Pariente J-L, Kim B-S, & Atala A (2002) In vitro biocompatibility evaluation of naturally derived and synthetic biomaterials using normal human bladder smooth muscle cells. *The Journal of urology* 167(4):1867-1871.

7. Yang H & Wright Jr JR (2002) Human β cells are exceedingly resistant to streptozotocin in vivo. *Endocrinology* 143(7):2491-2495.
